# Supplementary material for: Cell Migration in Endometriosis Responds to Omentum-Derived Molecular Cues Similar to Ovarian Cancer
Source: Int J Mol Sci. 2025 Feb 20;26(5):1822. doi: 10.3390/ijms26051822 (PMC11899264; doi:10.3390/ijms26051822)
Supplement: Supplementary file 1 [file ijms-26-01822-s001.zip › ijms-3446294-supplementary.pdf]

Supplementary Material

Table S1. List of primary cultures assayed in this study. This includes 18 tumors (16 ovarian cancer, 1 borderline mucinous tumor, 1 benign cyst), 8 endometriosis, and 29 omentum samples. Ovarian cancer subtypes were high grade serous carcinoma, clear cell carcinoma, endometrioid carcinoma, mucinous carcinoma. Abbreviation: NA, not available.

Sample	Histological type	Ovarian tumor / endometriosis culture	Omentum culture
KO20	High grade serous carcinoma	√	√
KO24	High grade serous carcinoma	√	√
KO27	High grade serous carcinoma	√	NA
E164	High grade serous carcinoma	√	NA
E187	High grade serous carcinoma	Non-viable	√
E208	High grade serous carcinoma	√	NA
E226	High grade serous carcinoma	Non-viable	√
E268	High grade serous carcinoma	√	√
E277	High grade serous carcinoma	√	√
E304	High grade serous carcinoma	NA	√
E317	High grade serous carcinoma	√	√
E335	High grade serous carcinoma	√	√
E340	High grade serous carcinoma	Non-viable	√
E343	High grade serous carcinoma	Non-viable	√
E231	Clear cell carcinoma	Non-viable	√
E233	Clear cell carcinoma	NA	√
E240	Clear cell carcinoma	√	√
E243	Clear cell carcinoma	√	√
E312	Clear cell carcinoma	NA	√
E338	Clear cell carcinoma	Non-viable	√
E404	Clear cell carcinoma	Non-viable	√
E406	Clear cell carcinoma	Non-viable	√
E255	Endometrioid carcinoma	√	√
E262	Endometrioid carcinoma	√	√
E337	Endometrioid carcinoma	√	√
E127	Mucinous carcinoma	√	NA
E261	Mucinous carcinoma	√	NA
E362	Mucinous carcinoma	Non-viable	√
E239	Borderline mucinous tumor	√	√
E329	Borderline mucinous tumor	Contaminated	√
KO34	Endometriosis	√	NA
KO48	Endometriosis	√	NA
E114	Endometriosis	√	NA
E128	Endometriosis	√	NA
E143	Endometriosis	√	NA
E195	Endometriosis	√	NA
E242	Endometriosis	NA	√
E271	Endometriosis	√	NA
E377	Endometriosis	Non-viable	√
E402	Endometriosis	√	√
E247	Benign cyst	√	√

Table S2. Transwell cell migration assays: fold change differences. Top panel: data details of Figure 2. Bottom panel: data details of Figure 5. Unpaired *t*-test was used for statistical comparison in the top panel. ANOVA followed by Tukey's multiple comparison test was used for statistical comparison in the bottom panel. For each experiment, five random fields were imaged per transwell and at least two replicates were included. PHA665752: c-MET inhibitor. *Abbreviations: Avg, average; HGSC, high grade serous carcinoma; CCC, clear cell carcinoma; EC, endometrioid carcinoma; MC, mucinous carcinoma; BorMT, borderline mucinous tumor; EMS, endometriosis.*

Cells in upper well	O-ASC CM in lower well	Avg no. of cells migrated in control (a)	Avg no. of cells migrated in O-ASC CM (b)	Avg no. of cells migrated in O-ASC CM + PHA665752 (c)	Fold Change (a vs b)	Fold Change (b vs c)	*p-value* (a vs b)	*p-value* (b vs c)
KO20 (HGSC)	KO20 (HGSC)	126	196	NA	1.50	NA	<0.0001	
E268 (HGSC)	E268 (HGSC)	40	145	NA	3.70	NA	<0.0001	
E335 (HGSC)	E335 (HGSC)	87	194	NA	2.20	NA	<0.0001	
E243 (CCC)	E243 (CCC)	91	223	NA	2.50	NA	<0.0001	
E255 (EC)	E255 (EC)	288	458	NA	1.60	NA	<0.0001	
E262 (EC)	E262 (EC)	156	334	NA	2.10	NA	<0.0001	
E337 (EC)	E337 (EC)	100	258	NA	2.60	NA	<0.0001	
E127 (MC)	E239 (BorMT)	207	320	NA	1.50	NA	<0.0001	
E261 (MC)	E239 (BorMT)	317	561	NA	1.80	NA	<0.0001	
KO48 (EMS)	E377 (EMS)	183	240	NA	1.30	NA	<0.0001	
E128 (EMS)	E377 (EMS)	170	268	NA	1.60	NA	<0.0001	
E402 (EMS)	E402 (EMS)	212	252	NA	1.20	NA	0.0098	
KO48 (EMS)	KO20 (HGSC)	61	140	NA	2.30	NA	<0.0001	
KO48 (EMS)	E243 (CCC)	72	162	NA	2.20	NA	<0.0001	
KO20 (HGSC)	KO20 (HGSC)	262	395	244	1.50	0.62	<0.0001	<0.0001
E268 (HGSC)	E268 (HGSC)	40	145	84	3.70	0.58	<0.0001	<0.0001
E335 (HGSC)	E335 (HGSC)	87	194	114	2.20	0.59	<0.0001	<0.0001
E255 (EC)	E255 (EC)	150	378	242	2.50	0.64	<0.0001	0.0002
E262 (EC)	E262 (EC)	316	583	449	1.80	0.77	<0.0001	<0.0001
E337 (EC)	E337 (EC)	123	318	183	2.60	0.58	<0.0001	<0.0001
E261 (MC)	E239 (BorMT)	76	317	160	4.20	0.50	<0.0001	<0.0001
KO48 (EMS)	E377 (EMS)	183	240	189	1.30	0.79	<0.0001	<0.0001
E128 (EMS)	E377 (EMS)	170	268	215	1.60	0.80	<0.0001	<0.0001

Table S3. List of omental-adipose stromal cells conditioned media (O-ASC CM) studied in the respective assays. O-ASC CM was derived from 11 high grade serous carcinoma, 8 clear cell carcinoma, 3 endometrioid carcinoma, 1 mucinous carcinoma, 2 borderline mucinous tumors, 3 endometriosis and 1 benign cyst.

Sample	Histological Subtype	Transwell migration assay	Wound healing assay	Luminex assay	NanoString	Angiogenesis assay	Proliferation assay
KO20	High Grade Serous carcinoma	✓	✓	✓	✓	✓	✓
KO24	High Grade Serous carcinoma			✓		✓	✓
E187	High Grade Serous carcinoma			✓			
E226	High Grade Serous carcinoma			✓		✓	
E268	High Grade Serous carcinoma	✓	✓	✓		✓	
E277	High Grade Serous carcinoma			✓			
E304	High Grade Serous carcinoma			✓			
E317	High Grade Serous carcinoma			✓			
E335	High Grade Serous carcinoma	✓	✓	✓		✓	
E340	High Grade Serous carcinoma			✓			
E343	High Grade Serous carcinoma			✓		✓	
E231	Clear cell carcinoma			✓		✓	
E233	Clear cell carcinoma			✓		✓	
E240	Clear cell carcinoma			✓		✓	
E243	Clear cell carcinoma	✓		✓		✓	
E312	Clear cell carcinoma			✓			
E338	Clear cell carcinoma			✓			
E404	Clear cell carcinoma			✓		✓	
E406	Clear cell carcinoma			✓		✓	
E255	Endometrioid carcinoma	✓	✓	✓		✓	
E262	Endometrioid carcinoma	✓	✓	✓		✓	
E337	Endometrioid carcinoma	✓	✓	✓	✓	✓	
E362	Mucinous carcinoma			✓		✓	
E239	Borderline mucinous tumor	✓	✓	✓		✓	✓
E329	Borderline mucinous tumor			✓		✓	
E242	Endometriosis			✓		✓	
E377	Endometriosis	✓	✓	✓	✓	✓	✓
E402	Endometriosis	✓	✓	✓		✓	
E247	Benign cyst			✓		✓	

Table S4. Cytokine profiles of omental-adipose stromal cells conditioned media (O-ASC CM) by Luminex assay: average, median and range. *Abbreviations: HGSC, high grade serous carcinoma; CCC, clear cell carcinoma; EC, endometrioid carcinoma; MC, mucinous carcinoma; BorMT, borderline mucinous tumor; EMS, endometriosis; BeCy, benign cyst.*

Histology	Descriptive	HGF	SDF-1a	MCP-1	VEGF-A	IL-6	IL-8	ENA-78	GROα	FGF-2	MMP-1	n
HGSC	Average (pg/ml)	235.36	1728.79	910.64	1944.22	1886.35	519.41	80.94	39.71	28.88	580.49	11
	Median (pg/ml)	97.81	993.78	830.88	1548.74	1900.35	308.28	48.85	26.61	6.09	441.21	
	Range (min - max)	15.11 - 877.99	191.06 - 5670.96	429.86 - 2024.94	602.31 - 3990.35	784.97 - 3310.40	42.81 - 2261.33	15.07 - 433.87	5.67 - 117.65	0.00 - 216.86	18.84 - 1743.67	
CCC	Average (pg/ml)	38.78	803.22	719.33	2890.19	2275.27	573.85	147.30	128.98	11.12	516.21	8
	Median (pg/ml)	31.88	561.315	746.03	1954.67	2035.60	395.38	68.95	37.20	5.18	398.78	
	Range (min - max)	7.39 - 99.71	9.53 - 2742.06	275.78 - 1155.97	598.16 - 12255.97	1262.78 - 4198.19	154.23 - 1787.02	20.70 - 735.51	10.88 - 749.91	0.00 - 49.73	75.21 - 1391.52	
EC	Average (pg/ml)	30.82	257.92	781.92	2197.47	2414.61	245.80	26.91	23.52	4.10	598.29	3
	Median (pg/ml)	31.07	292.07	628.93	2003.30	2246.22	242.51	21.73	17.32	4.67	568.06	
	Range (min - max)	18.64 - 42.75	0.00 - 481.69	584.89 - 1131.94	1367.72 - 3221.38	1628.21 - 3369.41	85.72 - 409.18	21.21 - 37.80	13.46 - 39.78	2.26 - 5.38	283.46 - 943.36	
MC	Average (pg/ml)	14.87	335.58	657.88	3111.47	1911.31	793.08	51.21	24.66	8.54	343.02	1
	Median (pg/ml)	14.87	335.58	657.88	3111.47	1911.31	793.08	51.21	24.66	8.54	343.02	
	Range (min - max)	NA	NA	NA	NA	NA	NA	NA	NA	NA	NA	
BorMT	Average (pg/ml)	29.42	984.03	558.21	1568.95	2713.98	1229.17	106.30	136.39	65.12	847.22	2
	Median (pg/ml)	29.42	984.03	558.21	1568.95	2713.98	1229.17	106.30	136.39	65.12	847.22	
	Range (min - max)	21.67 - 37.16	42.91 - 1925.15	551.41 - 565.01	581.61 - 2556.29	1501.16 - 3926.79	260.65 - 2197.68	16.99 - 195.61	10.35 - 262.42	1.47 - 128.76	105.79 - 1588.64	
EMS	Average (pg/ml)	43.38	593.47	766.55	1166.06	1600.55	893.36	33.90	24.39	5.21	570.20	3
	Median (pg/ml)	57.29	609.83	592.31	1115.83	1445.70	964.21	42.52	29.76	7.21	395.30	
	Range (min - max)	8.61 - 64.25	0.00 - 1170.59	382.23 - 1325.12	1107.79 - 1274.57	1072.26 - 2283.69	203.95 - 1511.91	14.23 - 44.94	9.01 - 34.40	0.50 - 7.93	150.22 - 1165.08	
BeCy	Average (pg/ml)	48.51	224.66	794.19	998.70	1070.34	498.92	26.25	14.22	0.00	729.48	1
	Median (pg/ml)	48.51	224.66	794.19	998.70	1070.34	498.92	26.25	14.22	0.00	729.48	
	Range (min - max)	NA	NA	NA	NA	NA	NA	NA	NA	NA	NA	

Table S5. List of differentially expressed genes between the migrated and unmigrated cell populations in ovarian cancer and endometriosis: fold change. *Abbreviations: HGSC, high grade serous carcinoma; EC, endometrioid carcinoma; EMS, endometriosis.*

Comparison	Gene	Log2FC	LinearFC	*p-value*
HGSC - Migrated vs Unmigrated (n = 4)	PTTG1	1.27	2.41	0.0116
	RBL1	1.14	2.21	0.0203
	MET	0.75	1.68	0.0172
	CNN1	0.68	1.60	0.0348
	HDAC5	-0.71	-1.63	0.0419
EC - Migrated vs Unmigrated (n = 3)	MAPKAPK3	2.06	4.18	0.0137
	NOTCH1	1.75	3.36	0.0132
	AGGF1	1.53	2.88	0.0216
	ID1	1.49	2.81	0.0079
	EPHA2	1.19	2.28	0.0020
	PTTG1	1.04	2.06	0.0116
	HDHD3	-1.75	-3.36	0.0216
EMS - Migrated vs Unmigrated (n = 3)	PTTG1	1.44	2.71	0.0123
	ARHGDIB	1.08	2.12	0.0419

Table S6. qRT-PCR: Ct values of *c-MET* and *GAPDH* in ovarian cancer, endometriosis and benign cyst.

Sample	Histological type	Ct values (*c-MET*)	Ct values (*GAPDH*)	Fold change
KO20	High grade serous carcinoma	25.60	16.82	7.23
KO24	High grade serous carcinoma	27.91	16.2	0.94
KO27	High grade serous carcinoma	25.36	15.92	4.55
E164	High grade serous carcinoma	26.25	16.57	3.83
E208	High grade serous carcinoma	24.2	15.95	10.39
E268	High grade serous carcinoma	26.12	16.84	5.11
E277	High grade serous carcinoma	25.66	15.85	3.55
E317	High grade serous carcinoma	23.98	16.79	21.67
E335	High grade serous carcinoma	23.81	16.27	16.97
E240	Clear cell carcinoma	22.28	17.39	106.5
E243	Clear cell carcinoma	26.26	16.45	3.52
E255	Endometrioid carcinoma	24.54	15.95	8.22
E262	Endometrioid carcinoma	28.00	15.86	0.7
E337	Endometrioid carcinoma	28.43	15.91	0.54
E127	Mucinous carcinoma	25.88	15.74	2.81
E261	Mucinous carcinoma	26.83	16.02	1.77
E239	Borderline mucinous tumor	26.41	16.4	3.06
KO34	Endometriosis	25.38	16.12	5.19
KO48	Endometriosis	24.43	16.47	12.72
E114	Endometriosis	25.64	16.09	4.21
E128	Endometriosis	26.35	16.00	2.42
E143	Endometriosis	25.81	15.32	2.21
E195	Endometriosis	26.37	15.97	2.36
E402	Endometriosis	24.51	16.01	8.78
E247	Benign cyst	27.58	15.95	1.00

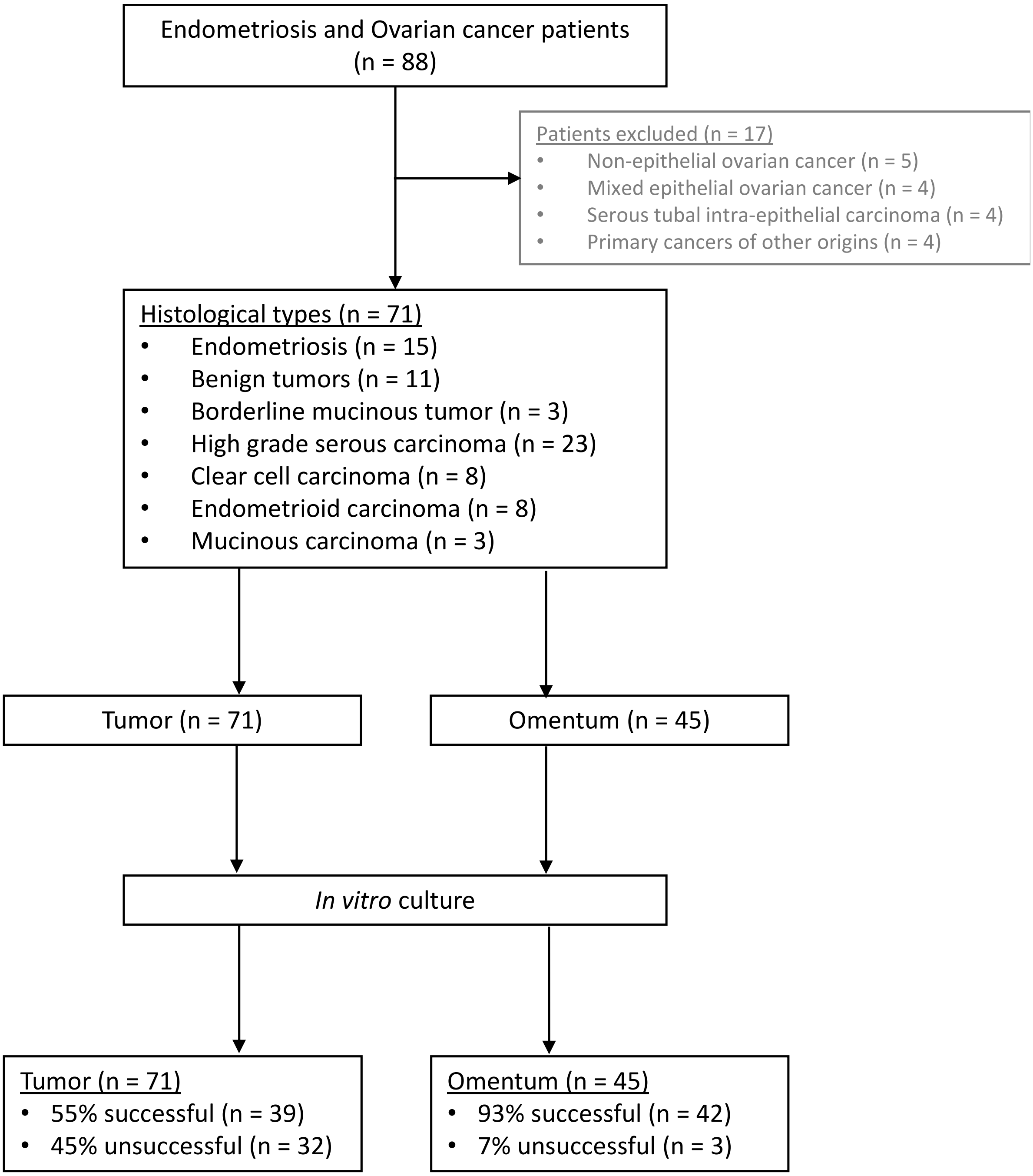

Figure S1. Study cohort of patients with endometriosis and ovarian tumors. Patients with endometriosis or ovarian tumors (malignant or benign) were recruited to the study. We excluded patients with non-epithelial ovarian cancer, mixed epithelial and non-epithelial ovarian cancer, pre-invasive serous tubal intra-epithelial carcinoma, other primary cancers with ovarian secondaries. Benign ovarian tumors include ovarian cyst, fibroma, thecoma, mucinous cystadenoma, serous cystadenofibroma. Expansion of these primary cell cultures beyond passage 2, with at least 3 vials of cryopreserved cells in storage, was considered successful. Unsuccessful cultures were those with insufficient starting number of viable cells, or terminated due to microbial contamination.

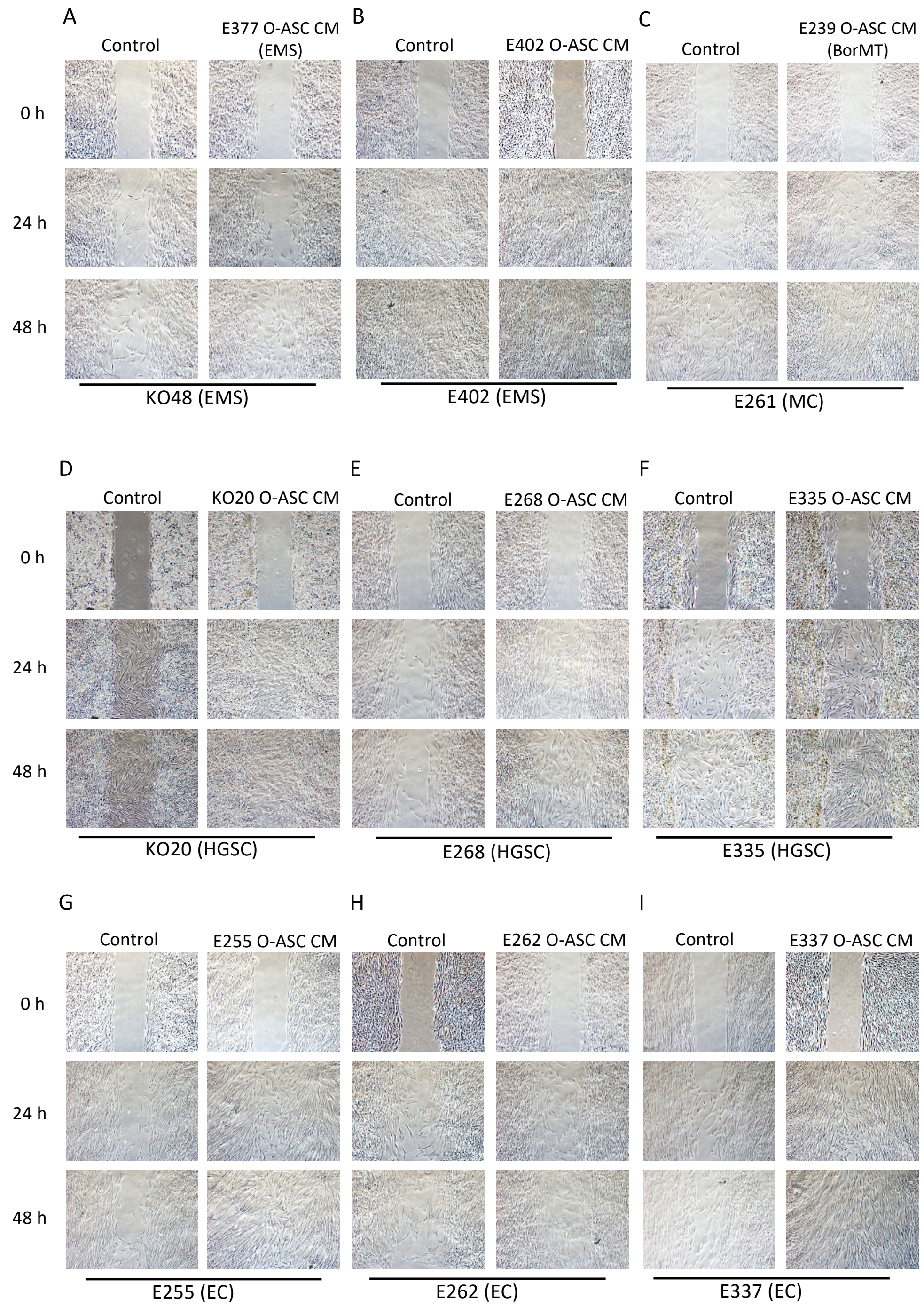

Figure S2. Wound healing assays confirmed that omental-adipose stromal cell conditioned media (O-ASC CM) increased cell migratory activity in primary cultures of endometriosis and ovarian cancer. O-ASC CM derived from ovarian cancer and endometriosis patients induced faster wound closure compared to control media. **(A-I)** For each assay, the bottom title denotes the type of cells studied, top panel labels show the media the cells were exposed to (either control media or O-ASC CM). Images of the wound area were captured at 0, 24, 48 hours, 40x magnification. **(A-B)** Wounds were induced in EMS (KO40, E402) and exposed to control media versus O-ASC CM derived from endometriosis patients E377 and E402, respectively. **(C)** Wounds were induced in MC (E261) and exposed to control media versus O-ASC CM derived from a BorMT patient. **(D-F)** Wounds were induced in HGSC (KO20, E268, E335) and exposed to control media versus O-ASC CM derived from the same patient. **(G-I)** Wounds were induced in EC (E255, E262, E337) and exposed to control media versus O-ASC CM derived from the same patient. *Abbreviations: HGSC, high grade serous carcinoma; EC, endometrioid carcinoma; MC, mucinous carcinoma; BorMT, borderline mucinous tumor; EMS, endometriosis.*

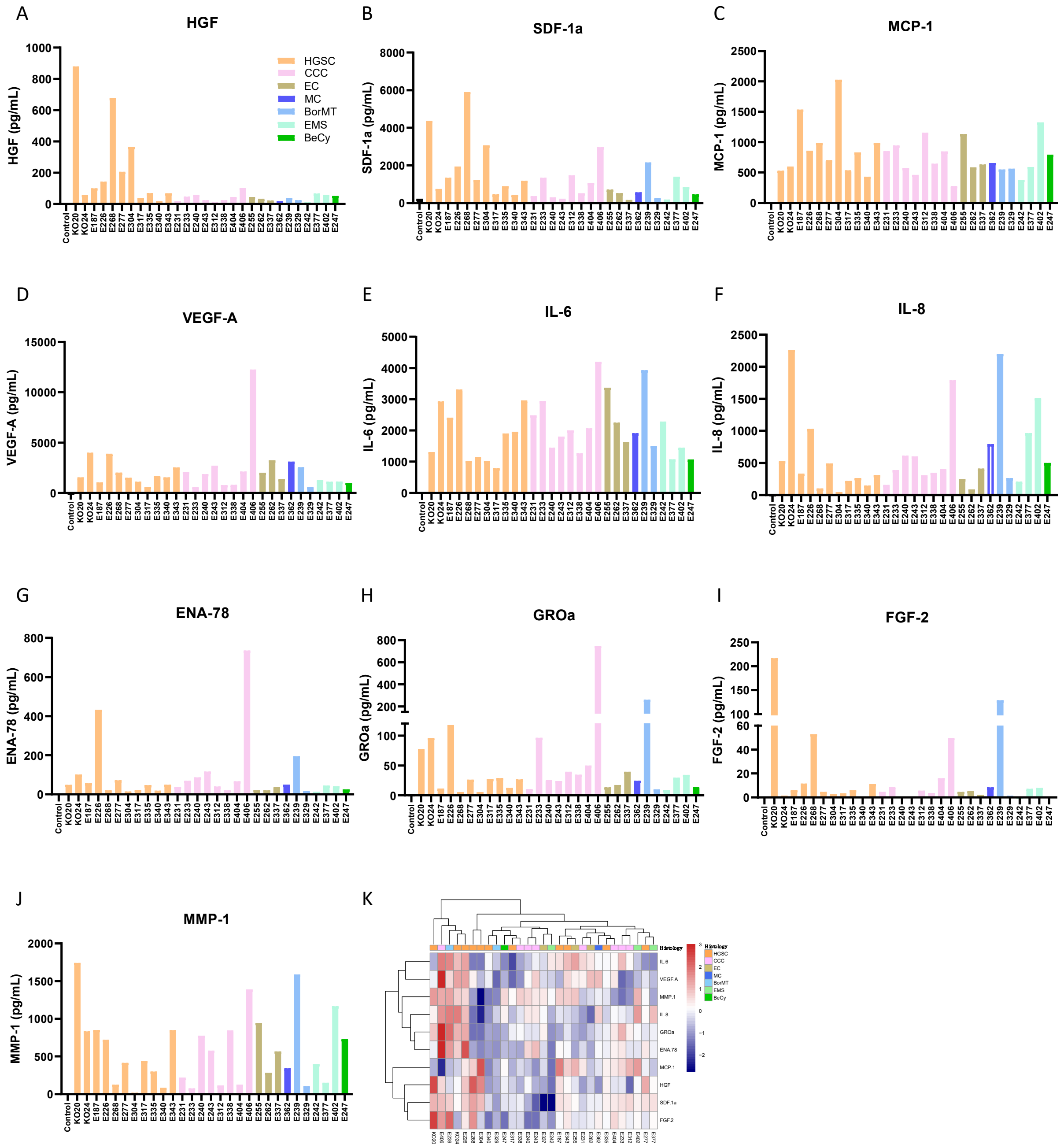

Figure S3. Cytokine profiles of individual omental-adipose stromal cell conditioned media (O-ASC CM) by Luminex assay. Quantification of **(A)** HGF, **(B)** SDF-1a, **(C)** MCP-1, **(D)** VEGF-A, **(E)** IL-6, **(F)** IL-8, **(G)** ENA-78, **(H)** GROa, **(I)** FGF-2 and **(J)** MMP-1 levels in O-ASC CM from patients with HGSC, CCC, EC, MC, BorMT, EMS and BeCy. **(K)** Heatmap showing the expression of these 10 cytokines in various histological types. *Abbreviations: HGSC, High grade serous carcinoma; CCC, Clear cell carcinoma; EC, Endometrioid carcinoma; MC, Mucinous carcinoma; BorMT, Borderline mucinous tumor; EMS, Endometriosis; BeCy, Benign cyst.*

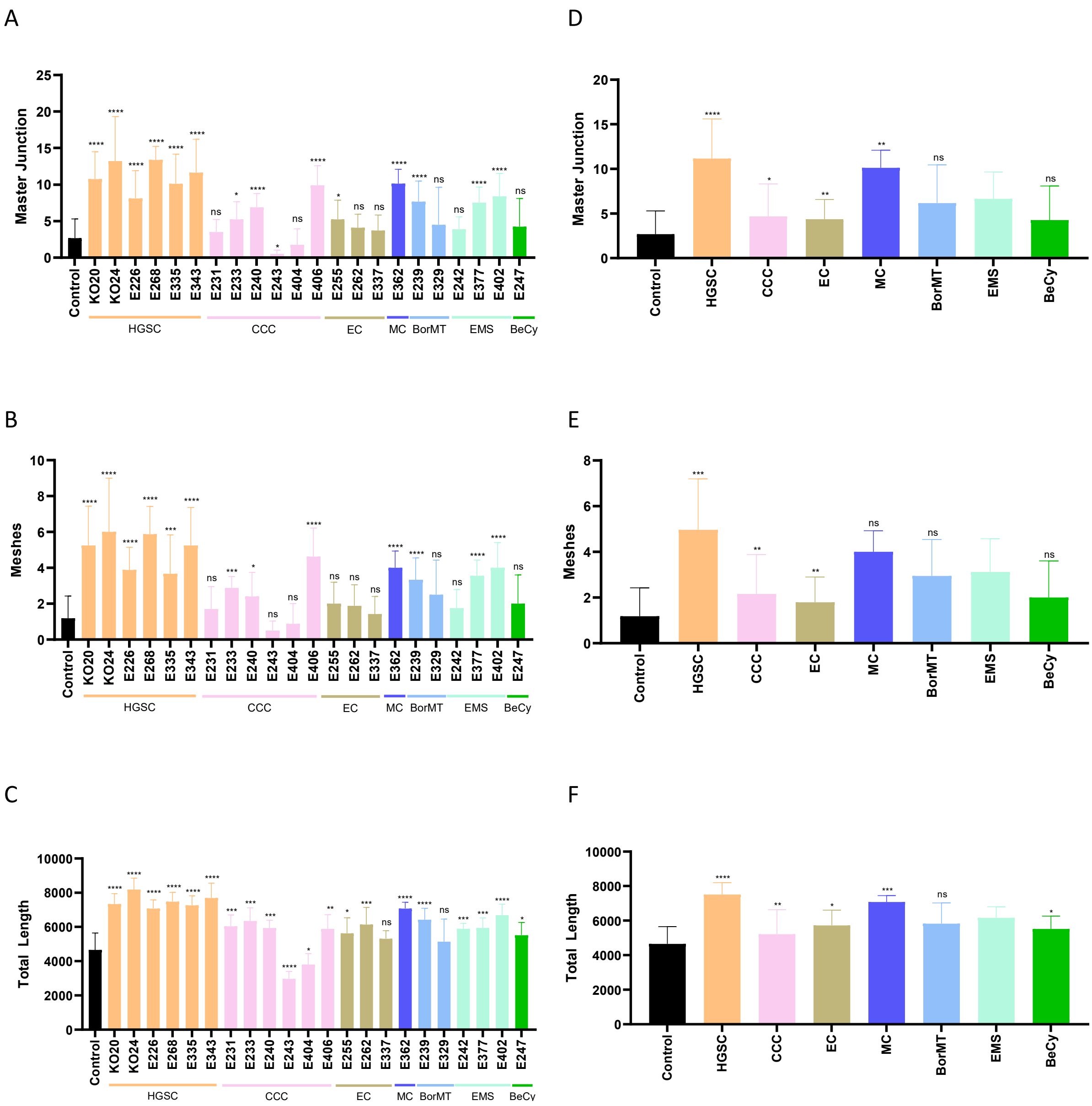

Figure S4. ***In vitro*** **angiogenesis: effect of omental-adipose stromal cell conditioned media (O-ASC CM) on tube formation in human umbilical vein endothelial cells (HUVECs).** Quantitative analysis of sprouting angiogenesis in a matrigel-based tube-forming assay, using the ImageJ Angiogenesis Analyzer to assess the parameters, namely number of master junctions, number of meshes and total tube length. **(A-C)** Effect of individual O-ASC CM on tube formation in HUVECs. For each parameter, Mann-Whitney U test was used for statistical comparison between each O-ASC CM and control. (**D-F)** Average effect of O-ASC CM on tube formation in HUVECs. For each parameter, Mann-Whitney U test was used for statistical comparison between each histological type and EMS. Results are shown as mean ± standard deviation (SD). For each experiment, five replicates were included. (NS: Not significant; * $p < 0.05$; ** $p < 0.01$; *** $p < 0.001$; **** $p < 0.0001$). *Abbreviations: HGSC, high grade serous carcinoma; CCC, clear cell carcinoma; EC, endometrioid carcinoma; MC, mucinous carcinoma; BorMT, borderline mucinous tumor; EMS, endometriosis; BeCy, benign cyst.*
